# Supplementary material for: Genomic and Pathological Characterization of Acute Hepatopancreatic Necrosis Disease (AHPND)-Associated Natural Mutant Vibrio parahaemolyticus Isolated from Penaeus vannamei Cultured in Korea
Source: Animals (Basel). 2024 Sep 26;14(19):2788. doi: 10.3390/ani14192788 (PMC11475263; doi:10.3390/ani14192788)
Supplement: Supplementary file 1 [file animals-14-02788-s001.zip › Table S2&S3(final).pdf]

Table S2. The T6SS-associated regions identified in the genome of *V. parahaemolyticus* strain 20-082A3 and 19-021-D1.

**T6SS***	***V. parahaemolyticus* 20-082A3**				***V. parahaemolyticus* 19-021-D1**			
	**Chromosome 1**		**Chromosome 2**		**Chromosome 1**		**Chromosome 2**	
	**Location (bp)**	**Identity (%)**	**Location (bp)**	**Identity (%)**	**Location (bp)**	**Identity (%)**	**Location (bp)**	**Identity (%)**
T6SS1	1270771-1302119	98.7	-	-	601989-63337	98.7	-	-
T6SS2	-	-	1624885-1650918	99.1	-	-	110727-136760	99.1

* The T6SS-associated regions were identified using the *V. parahaemolyticus* RIMD 2210633 genome sequence (NC_004603.1).

Table S3. Genomic Islands (GIs) identified in the genome of *V. parahaemolyticus* strain 20-082A3 and 19-021-D1.

GIs[*]	*V. parahaemolyticus* 20-082A3				*V. parahaemolyticus* 19-021-D1			
	Chromosome 1		Chromosome 2		Chromosome 1		Chromosome 2	
	Location (bp)	Identity (%)	Location (bp)	Identity (%)	Location (bp)	Identity (%)	Location (bp)	Identity (%)
VPaI-1	2450970-2493994	98.7	-	-	1781874-1824898	98.7	-	-
VPaI-2	2185924-2193740	98.4	-	-	1516828-1524644-	98.4	-	-
VPaI-3	1630583-1653798	98.5	-	-	961801-985016	98.5	-	-
VPaI-4	-	-	-	-	-	-	-	-
VPaI-5	-	-	-	-	-	-	-	-
VPaI-6	1428473-1455299	98.6	-	-	759691-786517	98.6	-	-
VPaI-7	1342671-1383867	98.1	-	-	673889-715085	98.1	-	-

[*] The GIs were identified using the *V. parahaemolyticus* RIMD 2210633 genome sequence (NC_004603.1).
