# Supplementary material for: Genomic and Pathological Characterization of Acute Hepatopancreatic Necrosis Disease (AHPND)-Associated Natural Mutant Vibrio parahaemolyticus Isolated from Penaeus vannamei Cultured in Korea
Source: Animals (Basel). 2024 Sep 26;14(19):2788. doi: 10.3390/ani14192788 (PMC11475263; doi:10.3390/ani14192788)
Supplement: Supplementary file 1 [file animals-14-02788-s001.zip › Table S5. Prophage (final).pdf]

Table S5. The four prophage regions detected in the genome of *Vp*$_{AHPND}$ mutant strain 20-082A3. The regions were determined using PHASTEST (https://phastest.ca/) and are shown along with their lengths, loci in the genome, direction, and completeness.

Region (Length, G+C %)	Completeness*	Most common phage / BLAST Hit	E-Value
**Chromosome 1**			
**Region I (31.6kb, 43.38%)**	**Incomplete**	**PHAGE_Lactob_phiAQ113_NC_019782**	
complement(905800..906969)		PP_00907;integrase family site-specific recombinase;phage;-;PHAGE_Pseudo_phi2_NC_030931	7.84e-50
complement(908012..908485)		PP_00911;hypothetical protein;phage;-;PHAGE_Pectob_ZF40_NC_019522	7.49e-11
complement(908532..911063)		PP_00912;putative DNA repair protein;phage;-;PHAGE_Pseudo_H66_NC_042342	1.46e-25
complement(912380..912736)		PP_00916;transcription regulatory protein prtR;phage;-;PHAGE_Escher_PA28_NC_041935	1.39e-12
complement(913337..914254)		PP_00918;hypothetical protein;phage;-;PHAGE_Bacill_vB_BhaS_171_NC_030904	1.17e-25
complement(914449..914904)		PP_00919;replication protein P;phage;-;PHAGE_Salmon_SEN34_NC_028699	4.79e-09
complement(914907..915272)		PP_00920;hypothetical protein;phage;-;PHAGE_Vibrio_X29_NC_024369	3.3e-12
complement(915515..916120)		PP_00922;hypothetical protein;phage;-;PHAGE_Entero_mEp237_NC_019704	1.06e-24
complement(916289..916690)		PP_00923;hypothetical protein;phage;-;PHAGE_Pseudo_MD8_NC_031091	2.19e-31
917235..917666		PP_00925;hypothetical protein;phage;-;PHAGE_Vibrio_nt_1_NC_021529	9.14e-05
complement(918349..919173)		PP_00928;putative endolysin;phage;-;PHAGE_Acinet_phiAC_1_NC_028995	2.17e-32
919733..920413		PP_00931;DNA methylase;phage;-;PHAGE_Psychr_pOW20_A_NC_020841	3.82e-34
920620..921549		PP_00932;phosphoadenosine phosphosulfate reductase;phage;-;PHAGE_Lactob_phiAQ113_NC_019782	2.51e-21
921600..922058		PP_00933;ectoine synthase/ParB multidomain protein;phage;-;PHAGE_Mycoba_Gaia_NC_026590	3.81e-30
924276..924764		PP_00937;putative terminase small subunit;phage;-;PHAGE_Pseudo_PaMx74_NC_028809	6.1e-10
924865..926184		PP_00938;gene 2 protein;phage;-;PHAGE_Shigel_Sf6_NC_005344	1.11e-18
**Region II (24.1Kb, 46.80%)**	**Intact**	**PHAGE_Burkho_BcepMu_NC_005882**	
complement(973220..973780)		PP_01005;C2;phage;-;PHAGE_Salmon_ST160_NC_014900	6.93e-05
973990..974211		PP_01006;transcriptional regulatory protein;phage;-;PHAGE_Mannhe_vB_MhM_3927AP2_NC_028766	1.29e-21
974232..976277		PP_01007;transposase;phage;-;PHAGE_Vibrio_12B12_NC_021070	5.38e-74

complement(976296..977225)		PP_01008;transposase B;phage;-;PHAGE_Bacill_BalMu_1_NC_030945	5.36e-11
complement(978612..979199)		PP_01013;hypothetical protein;phage;-;PHAGE_Pseudo_vB_PaeS_PM105_NC_028667	3.64e-26
complement(979209..979838)		PP_01014;hypothetical protein;phage;-;PHAGE_Pseudo_vB_PaeS_PAO1_Ab30_NC_026601	1.48e-23
complement(980214..980885)		PP_01016;hypothetical protein;phage;-;PHAGE_Burkho_phiE125_NC_003309	8.55e-11
complement(981770..981982)		PP_01018;hypothetical protein;phage;-;PHAGE_Vibrio_12B12_NC_021070	2.44e-24
complement(982133..983446)		PP_01019;hypothetical protein;phage;-;PHAGE_Entero_phiP27_NC_003356	6.98e-110
complement(983875..984273)		PP_01021;hypothetical protein;phage;-;PHAGE_Vibrio_12B12_NC_021070	4.51e-06
complement(985132..986031)		PP_01024;AB1gp73;phage;-;PHAGE_Acinet_AB1_NC_042028	1.22e-38
complement(986044..987180)		PP_01025;baseplate protein;phage;-;PHAGE_Bacter_Lily_NC_028841	2.19e-27
complement(987219..988232)		PP_01026;tail protein;phage;-;PHAGE_Burkho_BcepMu_NC_005882	1.13e-19
complement(990264..990827)		PP_01031;hypothetical protein;phage;-;PHAGE_Bacill_BalMu_1_NC_030945	1.54e-07
complement(991046..992674)		PP_01033;terminase large subunit;phage;-;PHAGE_Pseudo_H70_NC_027384	1.69e-37
992707..993489		PP_01034;putative baseplate protein;phage;-;PHAGE_Vibrio_X29_NC_024369	3.29e-16
complement(993612..993953)		PP_01035;phage baseplate assembly protein;phage;-;PROPHAGE_Salmon_Ty2	9.7e-05
complement(994036..995874)		PP_01036;tail protein;phage;-;PHAGE_Escher_vB_EcoM_ep3_NC_025430	2.52e-30
996402..997337		PP_01038;putative protease protein;phage;-;PHAGE_Pseudo_vB_PaeS_PM105_NC_028667	7.79e-06
**Chromosome 2**			
**Region III (25.2kb, 47.16%)**	**Intact**	**PHAGE_Escher_D108_NC_013594**	
698613..699545		**PP_00644;putative protease protein;phage;-;PHAGE_Pseudo_vB_PaeS_PM105_NC_028667**	7.54e-05
complement(700071..701912)		PP_00646;tail protein;phage;-;PHAGE_Escher_vB_EcoM_ECOO78_NC_041926	2.16e-28
complement(701990..702334)		PP_00647;phage baseplate assembly protein;phage;-;PROPHAGE_Salmon_Ty2	8.33e-05
702463..703254		PP_00648;putative baseplate protein;phage;-;PHAGE_Vibrio_X29_NC_024369	3.81e-14
703457..704932		PP_00650;hypothetical protein;phage;-;PHAGE_Bacill_BalMu_1_NC_030945	3.91e-23
705096..705656		PP_00651;hypothetical protein;phage;-;PHAGE_Bacill_BalMu_1_NC_030945	2.56e-10
707697..708698		PP_00657;tail protein;phage;-;PHAGE_Burkho_BcepMu_NC_005882	2.44e-20
708866..709873		PP_00658;baseplate protein;phage;-;PHAGE_Bacter_Lily_NC_028841	1.87e-36
709885..710781		PP_00659;putative endolysin;phage;-;PHAGE_Acinet_phiAC_1_NC_028995	5.36e-35
711616..712050		PP_00664;hypothetical protein;phage;-;PHAGE_Haemop_SuMu_NC_019455	1.56e-08
712175..713251		PP_00665;hypothetical protein;phage;-;PHAGE_Coryne_Juicebox_NC_048070	1.05e-93
713440..713586		PP_00666;hypothetical protein;phage;-;PHAGE_Vibrio_12B12_NC_021070	1.02e-15

713882..714868		PP_00668;hypothetical protein;phage;-;PHAGE_Aeromo_vB_AsaM_56_NC_019527	1.29e-19
715455..715823		PP_00670;hypothetical protein;phage;-;PHAGE_Vibrio_SIO_2_NC_016567	2.37e-21
716189..717229		PP_00672;putative nucleoid-associated protein;phage;-;PHAGE_Pseudo_H66_NC_042342	4.44e-29
717242..717829		PP_00673;hypothetical protein;phage;-;PHAGE_Mannhe_vB_MhM_3927AP2_NC_028766	3.19e-47
718756..719679		PP_00677;transposase B;phage;-;PHAGE_Bacill_BalMu_1_NC_030945	2.76e-14
719683..721707		PP_00678;transposase;phage;-;PHAGE_Vibrio_12B12_NC_021070	2.4e-71
721748..722023		PP_00679;Mu-like prophage FluMu DNA-binding protein Ner;phage;-;PHAGE_Haemop_SuMu_NC_019455	1.45e-17
722273..722968		PP_00680;putative cI repressor;phage;-;PHAGE_Aeromo_phiO18P_NC_009542	1.66e-21
723260..723883		PP_00682;gp245;phage;-;PHAGE_Bacill_G_NC_023719	3.02e-38
**Region IV (12.1Kb, 43.49%)**	**Intact**	**PHAGE_Vibrio_VEJphi_NC_012757**	
1781378..1781728		PP_01672;repressor;phage;-;PHAGE_Staphy_Ipla7_NC_018284	1.78e-11
1781858..1782937		PP_01673;RstA;phage;-;PHAGE_Vibrio_CTX_NC_015209	0.0
1782939..1783274		PP_01674;RstB;phage;-;PHAGE_Vibrio_CTX_NC_015209	1.82e-28
1783286..1783531		PP_01675;Vpf81;phage;-;PHAGE_Vibrio_Vf12_NC_005949	6.72e-40
1783539..1783772		PP_01676;putative major coat protein;phage;-;PHAGE_Vibrio_Vf33_NC_005948	5.63e-28
1783905..1785488		PP_01677;minor capsid protein;phage;-;PHAGE_Vibrio_VEJphi_NC_012757	5.05e-79
1785490..1785804		PP_01678;putative minor coat protein;phage;-;PHAGE_Vibrio_Vf33_NC_005948	1.0e-56
1785912..1786937		PP_01679;Vpf380;phage;-;PHAGE_Vibrio_Vf12_NC_005949	0.0
1787992..1788342		PP_01683;repressor;phage;-;PHAGE_Staphy_Ipla7_NC_018284	1.78e-11
1788472..1789551		PP_01684;RstA;phage;-;PHAGE_Vibrio_CTX_NC_015209	0.0
1789553..1789888		PP_01685;RstB;phage;-;PHAGE_Vibrio_CTX_NC_015209	1.82e-28
1789900..1790145		PP_01686;Vpf81;phage;-;PHAGE_Vibrio_Vf12_NC_005949	6.72e-40
1790153..1790386		PP_01687;putative major coat protein;phage;-;PHAGE_Vibrio_Vf33_NC_005948	5.63e-28
1790519..1792102		PP_01688;minor capsid protein;phage;-;PHAGE_Vibrio_VEJphi_NC_012757	5.05e-79
1792104..1792418		PP_01689;putative minor coat protein;phage;-;PHAGE_Vibrio_Vf33_NC_005948	1.0e-56
1792526..1793551		PP_01690;Vpf380;phage;-;PHAGE_Vibrio_Vf12_NC_005949	0.0

* The completeness of prophage regions was determined by the scores calculated from PHASTEST (https://phastest.ca/). [Intact (score > 90); Questionable (score 70-90); Incomplete (score < 70)].
