# Supplementary material for: Genomic and Pathological Characterization of Acute Hepatopancreatic Necrosis Disease (AHPND)-Associated Natural Mutant Vibrio parahaemolyticus Isolated from Penaeus vannamei Cultured in Korea
Source: Animals (Basel). 2024 Sep 26;14(19):2788. doi: 10.3390/ani14192788 (PMC11475263; doi:10.3390/ani14192788)
Supplement: Supplementary file 1 [file animals-14-02788-s001.zip › VpA3_Fig_S1&S2.pdf]

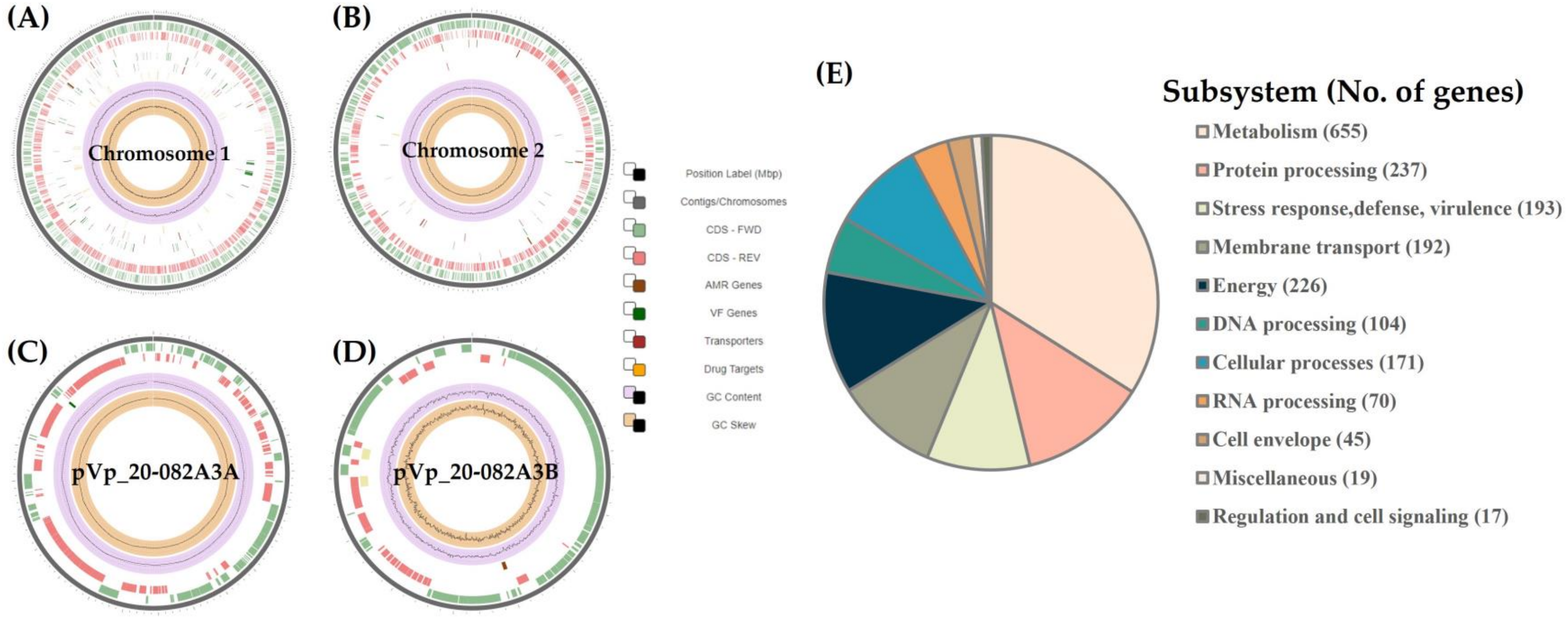

Figure S1. Genome maps of chromosome 1 (A), chromosome 2 (B), and two plasmids pVp_20-082A3A (C) and pVp_20-082A3B (D) from mutant *Vp*$_{AHPND}$ strain 20-082A3. The description of each track is presented as a legend on the right side. (E) Functional subsystem categories were generated based on the function of annotated CDSs in the whole genome of the strain 20-082A3. These categorizations and visualizations of CDSs were performed using the Pathosystems Resource Integration Center (PATRIC) v.3.6.12.

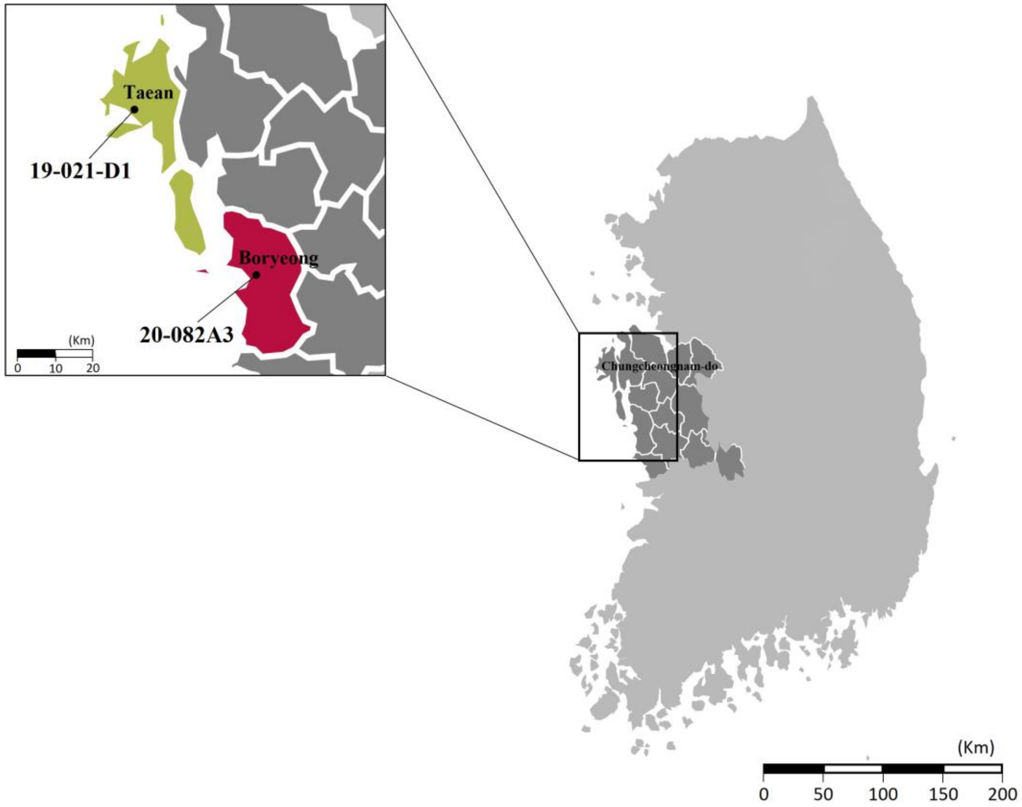

Figure S2. Geographical isolation sites of *Vp$_{AHPND}$* mutant strain 20-082A3 and the virulent strain 19-021-D1 in Korea. The map represents the geo graphical locations of the sampling sites for strain 19-021-D1 and strain 20-082A3. A light grey map indicates Korea, with the Chungcheongnam-d o province highlighted in dark grey. In the zoomed-in box of Chungcheongnam-do province indicates the specific isolation sites for each strain: T aean-gun (green) for strain 19-021-D1 and Boryeong-si (red) for strain 20-082A3.
